# Supplementary material for: “It Is Definitely a Good Program for Everyone from Every Community”: A Qualitative Study of Community Partner Perspectives on the Culturally and Linguistically Diverse (CALD) Mindfulness Program
Source: Int J Environ Res Public Health. 2023 Aug 18;20(16):6608. doi: 10.3390/ijerph20166608 (PMC10454450; doi:10.3390/ijerph20166608)
Supplement: Supplementary file 1 [file ijerph-20-06608-s001.zip › S1. Interview questions.docx]

Supplementary Materials

**S1. Interview questions**

| 1. What has been your involvement with the Culturally and Linguistically Diverse (CALD) Mindfulness Program?   – What was the main language of the community where you assisted?   1. Considering your involvement, can you comment on the CALD Mindfulness Program as a community wellbeing initiative?   – How the program has or hasn’t met the wellbeing needs of the community?   1. What impact do you think the program had:  - On the group participants? - On yourself? - On your organisation? - On the community in terms of understanding of mental health, stigma and access to relevant services?  1. In your opinion, what were the main factors that contributed to these outcomes? 2. As an employee of [partner organisation], how do you think your contribution has supported the delivery and influenced the program outcomes? 3. What were some of the challenges you encountered as a partner organisation or individual in your involvement in the program? 4. How did you overcome these challenges? – If they are ongoing, how could they be overcome? 5. Have there been groups in the community that missed out taking part in the program?  - If so, how do you think they can be reached?  1. What do you think is required to sustain such a program in the community? 2. In your opinion, should the program be continued, adjusted, expanded or curtailed?  - How could it be improved?  1. Would you be interested in continuing to your involvement with the program?  – If yes, in what capacity? 2. Any other comments or suggestions? |
| --- |
