# Supplementary material for: “It Is Definitely a Good Program for Everyone from Every Community”: A Qualitative Study of Community Partner Perspectives on the Culturally and Linguistically Diverse (CALD) Mindfulness Program
Source: Int J Environ Res Public Health. 2023 Aug 18;20(16):6608. doi: 10.3390/ijerph20166608 (PMC10454450; doi:10.3390/ijerph20166608)
Supplement: Supplementary file 1 [file ijerph-20-06608-s001.zip › S2. COREQ checklist.docx]

Supplementary Materials

S2. Consolidated criteria for reporting qualitative research (COREQ) 32-item checklist

| **Domain 1: Research Team and reflexivity** | |
| --- | --- |
| ***Personal Characteristics*** | |
| 1. Interviewer/facilitator | All interviews were conducted by Hanan Youssef (HY). |
| 2. Credentials | MPsych(Prof), BPsych(Hons) |
| 3. Occupation | Psychologist |
| 4. Gender | Female |
| 5. Experience and Training | Psychologist, Research Assistant  HY is a general psychologist with experience working across clinical populations in NSW Health, independent primary and secondary schools, and private practice. She gained research experience at UNSW Sydney and has worked on the CALD Mindfulness Program since its inception, assisting HS in the development, delivery and ongoing review. |
| *Relationship with participants* | |
| 6. Relationships established; 7. Participant knowledge of the interviewer | The Program is led by the SESLHD Multicultural Health Team in partnership with SESLHD and SLHD Mental Health Services, CESPHN, Western Sydney University (WSU) and community partners (organisations and individuals). This study involved the Multicultural Health Team, WSU and community partners.  HY was contracted by SESLHD to conduct the interviews in late 2021. Previously, she had co-facilitated three workforce development workshops with HS to train community partners (facilitators and co-facilitators) in program delivery. HY met four of the 16 interviewees during these 1-day workshops in 2017/18. HY also co-facilitated Arabic face-to-face mindfulness groups involving two other interviewees in 2017/18. HY had no prior connections with the other ten interviewees. |
| 8. Interviewer characteristics | As outlined in the answers to questions 1-6 above, Lebanese Australian. |
| **Domain 2: Study design** | |
| ***Theoretical framework*** | |
| 9. Methodological orientation and theory | As described in section 2.4.1 Design, our interpretive framework was based on pragmatism. We adopted an appreciative inquiry approach and a socio-ecological lens to explore both outcomes and process. |
| ***Participant selection*** | |
| 10. Sampling; 11. Method of approach; 12; Sample Size; 13. Non-Participation | The purposively-selected sample of community partners comprised bilingual mental health clinicians and community workers who had been involved in one or more of the face-to-face or online group programs for the Arabic or Bangla-speaking communities in the last two years (i.e., 2020-2021). Potential participants were initially contacted via email from HS.  From the 13 community partners (organisations and individuals), 17 people were identified as meeting the eligibility criteria: 16 were interviewed and one was unavailable. |
| ***Setting*** | |
| 14. Setting of data collection; 15. Presence of non-participants; 16. Description of sample | The telephone interviews were conducted in private at a time (and place) convenient for the participants. No non-participants were present during the interviews.  The final sample of 16 participants comprised 2 clinician facilitators and 14 community workers, 9 of whom had acted as co-facilitator. See section 3.1 Informant characteristics and Table 1 for further information. |
| ***Data collection*** | |
| 17. Interview guide; 18. Repeat interviews; 19. Audio/visual recording; 20. Field notes; 21. Duration; 22. Data saturation; 23. Transcripts returned | The semi-structured interview guide, which was piloted with one of the bilingual community workers, contained 12 open-ended questions. Questions were emailed to participants in advance and verbal consent was obtained at the start of the interview. The interviews were not recorded; however, HY took extensive notes to capture verbatim responses.  Interviews were approximately 30 minutes in length. There were no repeat interviews. Sixteen of the 16 of the 17 potential informants were interviewed; data saturation is not an issue this study.  Informants were given the opportunity to review their interview transcript. |
| **Domain 3. Analysis and findings** | |
| ***Data analysis*** | |
| 24. Number of data coders; 25. Description of the coding tree; 26. Derivation of themes; 27. Software; 28. Participant checking | The two data coders (HY and HS) were supervised by an experienced qualitative researcher (IB).  The qualitative data was examined following the Rigorous and Accelerated Data Reduction (RADaR) technique, which uses a team-based approach and general-purpose software to code and analyse qualitative data (Watkins, 2017)—See section 2.4.5 Analysis for details.  Tables were created in Microsoft Word. The thematic analysis was primarily semantic and combined inductive and deductive approaches. The responses of Arabic and Bangla informants were compared, and references to the clinical lead and trust/safety were coded, across the entire data set.  The whole team met regularly to reflect on each aspect of the research including the data analysis. Care was taken to ensure that the analysis accurately represented the views of the informants. No member checking with participants was conducted. |
| ***Reporting*** | |
| 29. Quotations presented; 30. Data and findings consistent; 31. Clarity of major themes; 32. Clarity of minor themes | The findings are supported by embedded quotes from all 16 participants. The results section is organised under six main headings: informant characteristics, perceived impact, contributing factors, community wellbeing, suggestions for improvement, and sustainability. Subheadings for perceived impact include group participants, community partner providers (informants themselves), community partner organisations, and community (broadly). |
